# Genome-wide analysis of Udai21: Unraveling the genetic basis of superior eating quality in rice

**DOI:** 10.1371/journal.pone.0324304

**Published:** 2025-11-25

**Authors:** Hiroki Ikeda, Masatsugu Tamura, Takayuki Ohnishi, Kenta Shirasawa

**Affiliations:** 1 School of Agriculture, Utsunomiya University, Utsunomiya, Tochigi, Japan; 2 Kazusa DNA Research Institute, Kisarazu, Chiba, Japan; ICAR - National Rice Research Institute, INDIA

## Abstract

Rice (*Oryza sativa* L.) is a staple for more than half of the world’s population, and grain quality strongly influences consumer preference and market value. Udai21, also referred to as Ohkome21, and previously designated as Yudai21, is a Japanese japonica cultivar noted for excellent eating quality and storage performance, yet its breeding origin and genomic basis remain unclear. Here, we aimed to resolve the genomic composition and likely parental origins of Udai21, and to identify candidate genes related to its distinctive quality traits. We generated a chromosome-scale assembly using PacBio HiFi long reads and assessed within-cultivar uniformity by sampling two breeder-maintained lines. We then profiled population structure using double digest restriction-site associated DNA sequencing together with representative accessions and conducted whole-genome resequencing and organelle genome analysis to trace ancestry, map polymorphisms, and annotate predicted impacts. Udai21 was predominantly japonica but carried introgressed aus-derived segments on chromosomes 1, 2, 3, 6, 7, and 10. Organelle single-nucleotide polymorphisms placed Udai21 with the aus cluster, consistent with an aus maternal origin and a japonica (Koshihikari) paternal contribution. The two maintenance lines showed high genetic uniformity. Among 37,522 predicted genes, we identified 1,017 non-redundant genes harboring high- or moderate-impact variants relative to Koshihikari, including loci previously implicated in grain quality and starch metabolism. These results support a hybrid origin for Udai21 (aus × Koshihikari) and provide a curated genome resource and candidate loci that can enable marker-assisted selection and informed crossing to combine superior eating quality with postharvest stability.

## Introduction

Rice (*Oryza sativa* L.) is a staple food for more than half of the global population [1,2], and grain quality—particularly eating and cooking quality—strongly influences consumer preference and market value [3,4]. According to data from the Food and Agriculture Organization of the United Nations (http://faostat.fao.org/), global rice production in 2023 was approximately 799.9 million tons, about 90% of which was produced in Asia [5]. Globally recognized premium long-grain cultivars such as Basmati, Jasmine, and Thai Hom Mali illustrate how specific sensory attributes (such as extra-long slender grains; exquisite aroma; sweet, tender texture; lengthwise elongation during cooking with minimal lateral swelling) define high quality in their respective markets [6–8]. In East Asia (e.g., Japan, Korea, northern and central China), short-grain japonica varieties dominate consumer demand [9], and Japan is estimated to rank around 12th in global rice production in recent statistics (http://faostat.fao.org/).

In Japan, Koshihikari has been the most extensively cultivated variety since 1979, occupying approximately 33% of the planting area in 2023, combining high eating quality with favorable agronomic traits, including adaptation to diverse environments, resilience to pre-harvest sprouting, and cold tolerance at booting [10]. However, the expansion of other high-quality and high-yielding cultivars has led to a gradual decline in its national share. Within this context, Udai21 represents a rare rice variety developed at the University Farm of the School of Agriculture, Utsunomiya University, Japan, in 1990, and registered in January 2010. In recent years, Udai21 has drawn attention for its scientifically proven exceptional taste and won numerous awards at national rice taste contests. It is characterized as a suitable variety for both fresh and stored grains, with higher eating quality (higher adhesiveness and adhesion) and lower starch digestibility than those of the Koshihikari and Milky Queen varieties, regardless of storage duration [11]. Further studies revealed that the texture of Udai21 was sensitive to the water-to-rice ratio (W/R ratio), showing the highest adhesiveness at a W/R ratio of ×1.6 and a lower syneresis rate than that of Koshihikari during post-cooking storage [12]. These findings indicate that Udai21 offers both high palatability and health benefits.

As living standards rise and the global rice trade expands, demand for high-quality rice continues to grow [13,14]. Therefore, developing new cultivars with desirable attributes—particularly appearance and eating quality—that determine market value is an urgent priority [14]. In this context, Udai21, noted for its high palatability and potential health benefits, represents both a compelling candidate for wider deployment and a valuable donor parent for breeding programs aiming to integrate superior eating quality with enhanced postharvest and storage stability. However, grain quality is inherently multifactorial, comprising multiple component attributes, each controlled by numerous interacting genes [13,14].

Moreover, the breeding origin—including parental contributions, cytoplasmic lineage, and the genomic basis underpinning Udai21—remains unresolved. Regarding its breeding history, a faculty member at Utsunomiya University reportedly identified, in 1990, an individual plant in the experimental paddy fields that bore conspicuously larger panicles than its neighbors. Through successive cycles of cultivation and selection among lines exhibiting superior eating quality, an individual with exceptionally large panicles and a plant height exceeding typical plants by more than 10 cm was isolated. This line was subsequently registered as a cultivar in 2010. However, detailed records from 1990—such as which cultivars were grown concurrently in the same field—are unavailable, and the breeding process has not been documented in a peer-reviewed publication. Whether introgressed segments from non-japonica groups contribute to its phenotype or whether the cultivar remains genetically uniform across maintained lines is unknown. This research gap constrains rational cross-design aimed at combining quality with resilience, limits marker-assisted selection anchored in causative regions, and complicates genetic stewardship of cultivar identity and seed purity.

In this study, we aimed to define the genomic composition and probable parental origins of Udai21, and to identify candidate genes associated with its distinctive traits. We hypothesized that Udai21 was predominantly japonica but harbored introgressed regions that contribute to quality-related functions. To test this, we (i) generated a chromosome-scale genome assembly using PacBio HiFi long reads, (ii) assessed within-cultivar genetic structure through double digest restriction-site associated DNA sequencing (ddRAD-Seq) alongside representative rice accessions, and (iii) performed whole-genome resequencing to trace ancestry, map polymorphisms, and annotate putative functional effects. By resolving the genomic background and highlighting candidate loci, this study provides a reproducible genomic resource and a practical foundation for breeding programs aiming to optimize eating quality and storage adaptability in rice.

## Materials and methods

### Genome assembly and gene prediction

Because Udai21 has been maintained as multiple breeder lines advanced through selfing and selection from the progenitor line, we sampled 10 individuals from two maintenance lines (#6 and #7; five plants each) to verify genetic identity. Genomic DNA was isolated from young leaves using Genomic-tips (Qiagen, Hilden, Germany). The genomic DNA from Udai21 #7−1 was subjected to long-read library preparation using the SMRTbell Express Template Preparation Kit 2.0 (PacBio, Menlo Park, CA, USA). The library was sequenced with an SMRT Cell 8M and the Sequel Sequencing Kit 2.0 on a Sequel II system (PacBio). The obtained HiFi reads were assembled into contigs using Hifiasm [15]. Organelle-derived sequences were removed by BLAST [16] searches against the plastid and mitochondrial genomes of Nipponbare (NC_001320.1 and NC_011033.1). The remaining contigs were aligned to the chromosome sequence of Nipponbare, Os-Nipponbare-Reference-IRGPS-1.0 [17] using RaGOO [18] and concatenated with 100 Ns to generate a chromosome-scale genome assembly for Udai21. Protein-coding genes were predicted using the MAKER pipeline [19]. Sequence completeness was evaluated using the BUSCO software [20]. Telomere repeats (TTTAGGG) were detected using a Telomere Identification toolkit [21].

### Genetic diversity analysis with ddRAD-Seq

Genomic DNA isolated from 10 individuals was digested with PstI and MspI to generate the ddRAD-Seq library, following the procedure described previously [22]. The library was sequenced on a DNBSEQ-G400 platform (MGI Tech, Shenzhen, China) in paired-end 100 bp mode. Additionally, ddRAD-Seq reads from the World Rice Core Collection [23] (obtained from a public database [24]) were used. High-quality reads were selected by trimming adapters with fastx_clipper (parameter, -a AGATCGGAAGAGC) in the FASTX-Toolkit (http://hannonlab.cshl.edu/fastx_toolkit) and deleting low-quality bases using PRINSEQ [25]. Reads were aligned on the chromosome-scale genome assembly of Udai21 using Bowtie2 [26] v.2.2.3, and sequence variants were detected with the mpileup command in SAMtools [27]. High-confidence single-nucleotide polymorphisms (SNPs) were selected using VCFtools [28] (parameters: --minDP5 --minQ 999 --max-missing 0.75). Population structure was evaluated through maximum-likelihood estimation of individual ancestries using ADMIXTURE [29] and principal component analysis with TASSEL 5 [30]. Genetic distances among the tested lines were calculated using the neighbor-joining method, and a dendrogram was generated in TASSEL 5 [30].

### Ancestry estimation with whole-genome sequencing analysis

A whole-genome shotgun (WGS) library was prepared from 10 individuals using the Swift 2S Turbo DNA Library Kit (Swift Biosciences, Ann Arbor, MI, USA) and sequenced on a DNBSEQ-G400 platform (MGI Tech) in paired-end 150 bp mode. Together with the obtained reads, WGS data for the World Rice Core Collection [31] and Koshihikari [24] (obtained from a public database) were used. High-quality reads were selected by trimming adapters with fastx_clipper (parameter, -a AGATCGGAAGAGC) in the FASTX-Toolkit (http://hannonlab.cshl.edu/fastx_toolkit) and deleting low-quality bases using PRINSEQ [25]. Reads were aligned on the chromosome-scale genome assembly of Udai21 and the organelle genome sequences of Nipponbare using Bowtie2 [26]. Sequence variants were detected using the mpileup command in SAMtools [27], and high-confidence SNPs were selected using VCFtools [28] (parameters --minDP5 --minQ 999 --max-missing 0.75). SNP density for every 100-kb genome segment was calculated using VCFtools [28]. SNP effects on gene function were estimated using SnpEff [32], and gene functions were presumed by referring to the OGRO database [33].

## Results

### Genome assembly and gene prediction

A total of 22.6 Gb HiFi reads were obtained for Udai21 (#7−1), with an N50 read length of 31.1 kb. The reads were assembled into 132 contigs with an N50 length of 30.0 Mb, yielding a total assembly size of 387.7 Mb. Potential organellar genome sequences (6.3 Mb from 93 contigs) were removed. The remaining 39 contigs (381.4 Mb length; N50 length, 30.0 Mb; Table 1) achieved a complete BUSCO score of 98.7% (Table 2) and were aligned to the Nipponbare reference genome sequence (Os-Nipponbare-Reference-IRGPS-1.0) to generate 12 chromosome-scale sequences spanning 381.4 Mb. This assembly was designated as UD21_r1.0 (Table 3). Telomere repeats were found at both ends of the 12 pseudomolecular sequences (Table 3).

**Table 1:** Genome assembly statistics for Udai21.

	Genome assembly
No. of contigs	39
Contig length (bp)	381,395,677
Contig N50 (bp)	30,030,604
No. of genes	37,522

**Table 2:** Genome assembly and predicted gene BUSCO scores.

	Genome assembly	Predicted genes
Complete BUSCOs	98.7%	96.1%
Complete single-copy BUSCOs	96.6%	94.1%
Complete duplicated BUSCOs	2.1%	2.0%
Fragmented BUSCOs	0.8%	2.4%
Missing BUSCOs	0.5%	1.5%

**Table 3:** Statistics for Udai21 pseudomolecule sequences.

Chromosome	Sequence length	No. of contigs	No. of genes
UD21ch01	43,811,085	2	4,990
UD21ch02	36,325,143	2	4,055
UD21ch03	37,516,008	2	4,413
UD21ch04	35,472,625	1	3,273
UD21ch05	30,396,699	1	3,049
UD21ch06	32,186,226	1	3,115
UD21ch07	30,030,604	1	2,868
UD21ch08	28,576,875	1	2,655
UD21ch09	25,601,763	23	2,170
UD21ch10	23,964,801	1	2,155
UD21ch11	29,976,136	3	2,549
UD21ch12	27,540,412	1	2,230
**Total**	**381,398,377**	**39**	**37,522**

A total of 101,205 potential protein-coding genes were predicted, of which 37,522 genes were selected as high-confidence genes with AED scores ≤0.5 and lengths of >180 bases. The complete BUSCO score for the high-confidence genes was 96.1% (Table 2).

### Genetic diversity analysis

**Fig 1:**
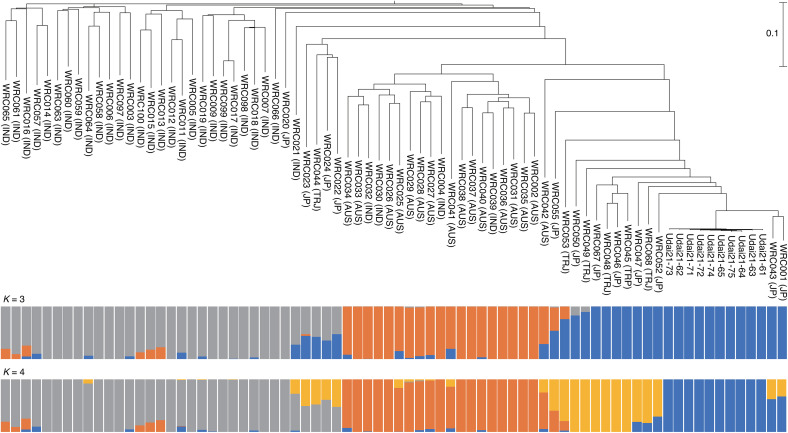
Classification of Udai21 and the World Rice Core Collection based on ddRAD-Seq data. Top: Dendrogram of the rice lines. JP, TRJ, AUS, and IND indicate *temperate japonica*, *tropical japonica*, *aus*, and *indica* varieties, respectively. Middle and bottom: Admixture charts for classes 3 and 4.

**Fig 2:**
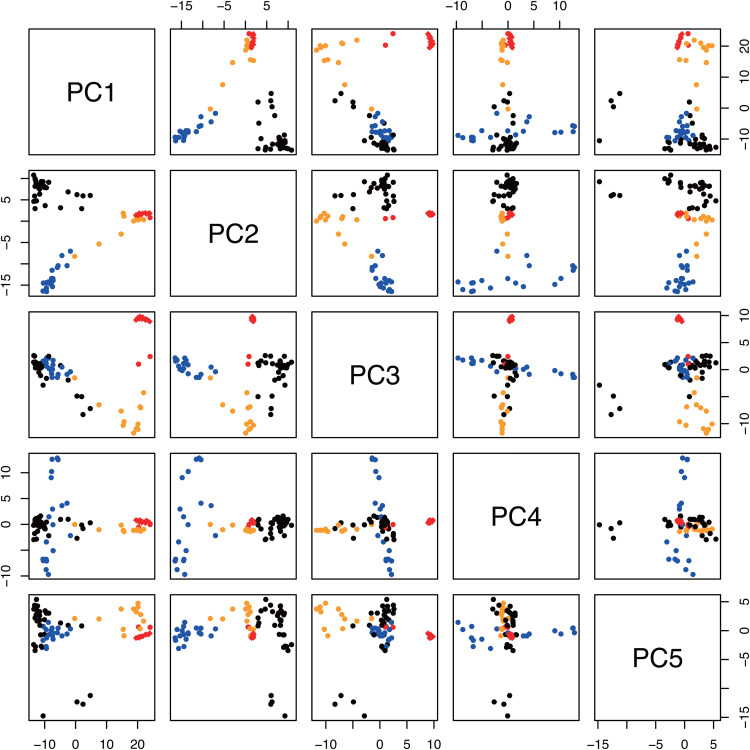
Principal component analysis of Udai21 and the World Rice Core Collection. Red, orange, blue, and black dots indicate *temperate japonica*, *tropical japonica*, *aus*, and *indica*, respectively.

ddRAD-Seq reads were obtained from 10 samples of Udai21 and 69 lines of the World Rice Core Collection from a public DNA database. The reads were mapped onto UD21_r1.0 to detect 4,000 high-quality SNPs. Based on the SNPs, the tested 69 lines were grouped into three subpopulations: japonica, indica, and aus (Fig 1). The 10 individuals of the two Udai21 lines were classed, as expected, in the japonica group and were closely related to each other, indicating that the Udai21 lines were genetically identical. This classification was supported by the admixture and principal component analyses (Figs 1 and 2), which indicated that Udai21 was genetically close to Nipponbare and Dianyu 1 as japonica members, among the tested lines.

### Estimation of Udai21 ancestry

**Fig 3:**
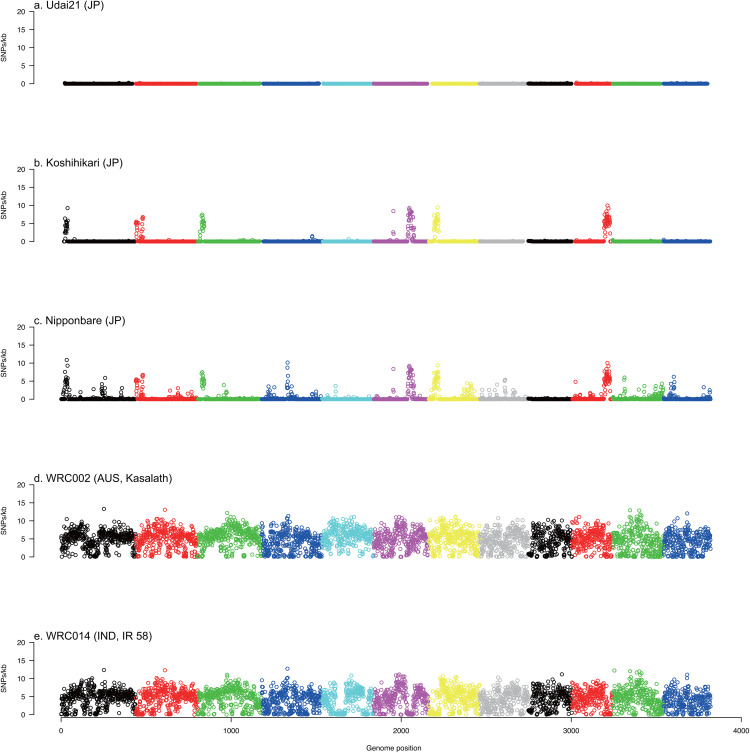
Rice chromosome SNP density for five rice varieties. Dots indicate SNP density in a 100-kb window size for Udai21 (a), Koshihikari (b), Nipponbare (c), Kasalath (d), and IR 58 (e). Colors indicate the 12 chromosomes of the rice genome.

WGS reads for 10 individuals of Udai21, 69 lines of the World Rice Core Collection, Nipponbare, and Koshihikari were mapped onto UD21_r1.0 to detect 3,549,925 high-quality SNPs. In the Udai21 samples, 100% of the SNP genotypes matched the reference sequences, as expected (Fig 3a). Koshihikari (97.7%) and Nipponbare (96.2%) exhibited scores slightly lower than that of Udai21. Interestingly, SNP clusters were found in Koshihikari at the short arms of chromosomes 1, 2, 3, 7, the long arm of chromosome 10, and the middle of chromosome 6 with an SNP density of 10 SNPs/1 kb (Fig 3b). In addition to Koshihikari, Nipponbare had more SNPs on other chromosomes except chromosome 9 (Fig 3c). Conversely, the aus variety Kasalath and the indica variety IR 58 had only 47.7% and 50.9% reference alleles, respectively (Fig 3d and e). SNPs in Kasalath were distributed over the genome with an SNP density of 10 SNPs/1 kb (Fig 3d).

**Fig 4:**
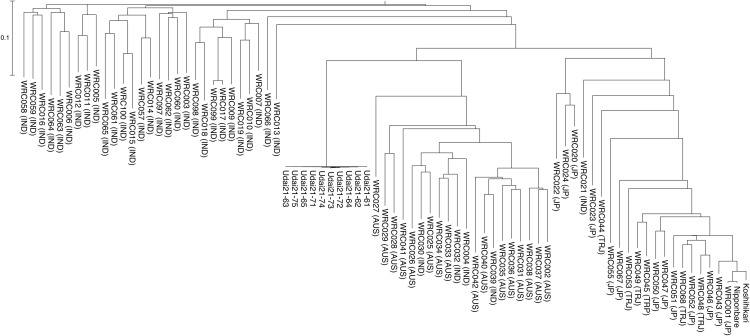
Classification of Udai21 and the World Rice Core Collection based on whole genome sequencing data.

**Fig 5:**
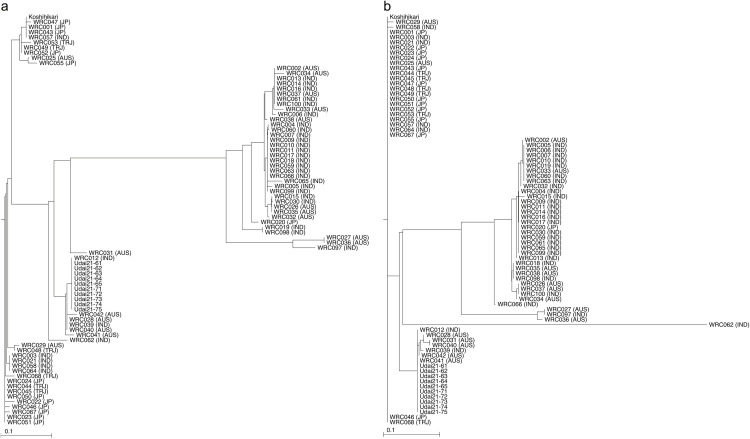
Classification of Udai21 and the World Rice Core Collection based on organelle genome data. Dendrograms are based on plastid (a) and mitochondrion (b).

Next, the tested lines were clustered based on the SNP genotypes in the SNP clustered regions. Three major subpopulations (japonica, indica, and aus) were expectedly formed as above; however, Udai21 was joined to aus (Fig 4). Subsequently, the WGS reads were mapped on the organelle genome sequences of Nipponbare to detect 105 and 94 SNPs. Based on the SNP genotypes, the tested lines were grouped into the three subpopulations and Udai21 was joined to the aus cluster (Fig 5a and b).

### Genes that comprise Udai21

Among the 37,522 genes predicted in the Udai21 genome, 3,323 genes were located at the SNP clusters. High-impact and moderate SNPs showing polymorphisms between Udai21 and Koshihikari were found in 92 and 1,007 genes, respectively, of which 82 genes overlapped. Of the 1,017 non-redundant genes (S1 Table), 30 were functionally annotated as genes for resistance or tolerance (nine genes), physiological traits (20 genes), and morphological traits (eight genes) (Tables 4 and S2). Note that some genes are assigned to more than one category; therefore, the summed counts across the three categories exceed 30. For example, UD21ch02g05358 is annotated under both the major categories Resistance or Tolerance and Physiological trait, and further classified into the minor categories Salinity tolerance, Blast resistance, and Others. Full, literature-based annotations for all loci are provided in S2 Table.

**Table 4:** Category-level summary of genes with potentially distinct functions between Udai21 and Koshihikari.

Trait (Major category)	Trait (Minor category)	No. of genes
Resistance or Tolerance	Blast resistance	5
	Bacterial blight resistance	2
	Salinity tolerance	2
	Drought tolerance	1
	Lodging resistance	1
	Other soil stress tolerance	1
	Other stress resistance	1
Physiological traits	Sterility	8
	Flowering	5
	Eating quality	4
	Germination dormancy	2
	Others	2
	Source activity	2
	Lethality	1
	Root	1
Morphological traits	Dwarf	4
	Culm leaf	2
	Root	2
	Seed	2
	Panicle flower	1
	Shoot seedling	1
Others		3

Note: Counts reflect the number of genes annotated per category; full gene-by-gene details and references are provided in S2 Table.

## Discussion

Wheat, rice, maize, and soybeans provide two-thirds of human caloric intake worldwide [34], with over half of the global population relying on rice as a main food source [1,2]. Crops are sensitive to climate change, including changes in temperature and precipitation, and rising atmospheric CO_2_ concentrations. Among these factors, temperature increase has the most negative impact on crop yield [34]. Field warming experiments produce substantial losses in rice yield. Without CO_2_ fertilization, effective adaptation, and genetic improvement, severe rice yield losses are plausible under intensive climate warming scenarios [2]. Global warming has a significant impact on crop productivity and quality [35].

Among the most intensively cultivated grains, rice is consumed as a whole grain. The texture of rice is influenced by the structural properties of the cell walls and tissues of the grain, and the characteristics of rice starch play a significant role in consumer preference. For example, the japonica variety is highly preferred due to its soft and sticky texture [36]. However, rising global temperatures can potentially increase the incidence of white immature grains and reduce yields, in addition to adversely affecting the stickiness of cooked rice. Rice grain quality is influenced by multiple factors, such as physical appearance, eating and cooking qualities, and nutritional content [3]. Specifically, the eating and cooking qualities of rice are key considerations for buyers and therefore significantly influence rice market price, consumer acceptance, and breeding efforts to improve grain quality [4]. Consequently, the development of rice varieties that maintain grain quality while maintaining heat tolerance has become a crucial strategy for mitigating the effects of climate change.

Since its development in 1956, Koshihikari has remained the most important and widely grown rice cultivar in Japan [10]. However, the cultivar faces several production challenges, including weak resistance to leaf blast, slightly weak resistance to panicle blast, and susceptibility to leaf stripe virus [10]. Moreover, a decline in quality due to heat stress has been observed in many prefectures [10]. The cultivar Udai21 shows fewer occurrences of white immature grains under high-temperature cultivation conditions, higher adhesiveness and cohesion [11], and lower retrogradation after refrigeration [12] than does Koshihikari. Because grain quality strongly influences both farmer’s and consumer’s varietal choices [37], cultivating varieties like Udai21 that maintain superior flavor under high temperatures—or using them as donor parents in breeding programs targeting high eating quality under heat—can be advantageous. However, as noted in the introduction, detailed breeding records for Udai21 are not available, and its genetic background has remained unknown. Consequently, this study aimed to employ next-generation sequencing and genomic analysis techniques to elucidate the progenitors and origins of Udai21.

Genome sequencing analysis indicated that the genomic background of Udai21 includes Koshihikari and possesses at least six genome fragments introgressed from aus-type lines (Fig 3a). Aus-type rice is closely related to indica-type rice and constitutes a distinct genetic group [38]. Aus-type accessions such as Kasalath harbor higher genomic diversity than the japonica variety Nipponbare [39], and this type is generally characterized by early maturity, short stature, environmental stress tolerance, and summer cultivation [40]. For example, the submergence tolerance gene *OsSUB1A* and phosphorus starvation tolerance gene *OsPSTOL1* have both been identified from aus-type rice varieties [41,42]. Notably, Aus-type variety N22 is currently one of the most heat-tolerant rice cultivars [43,44]. The Dular variety has shown the highest P uptake under low-P field conditions [45] and has been ranked highest in a drought study, with a yield reduction lower over multiple seasons than that of other genotypes [46]. In addition, the genotype group mainly consisting of aus-type indica tended to have higher stomatal conductance to CO_2_, whereas the genotype group corresponding to japonica had a higher nitrogen concentration in leaves [40]. Near-isogenic lines and chromosome segment substitution lines derived from Kasalath and Nipponbare have enabled the cloning and functional identification of genes underlying heading date, grain size, plant height, seed shattering, and seed dormancy [47–51]. Therefore, aus-type rice is highly valuable for breeding applications as a source of novel tolerance traits and for gene discovery research [52]. Although we found no prior studies directly linking aus-type genomes to improved eating quality per se, experience from horticultural crops such as tomato showed that superior flavor attributes can arise from more distantly related wild relatives rather than only elite cultivars [53]. These observations are consistent with the hypothesis that the superior traits of Udai21 (e.g., eating quality) are partly attributable to aus-derived genomic segments.

As noted in the Introduction, the progenitor line of Udai21 was identified in 1990 from rice cultivated in experimental paddy fields at the University Farm of the School of Agriculture, Utsunomiya University, Japan. Annual selection of only high-quality lines led to the development of Udai21, which has recently garnered national recognition for its eating quality in multiple taste competitions. However, the origins, parent varieties, and lines have remained unknown, with the assumption that Udai21 was a mutant of Koshihikari. This study revealed that the progenitor line of Udai21 results from a cross between Koshihikari and an aus-type rice. Since the organelle genomes of Udai21 were also of the aus type (Fig 5a and b), we propose that the ancestor of Udai21 was generated from a cross between an aus line as the maternal line and Koshihikari as the paternal line. Thereafter, the line was advanced through selfing and selection to develop Udai21. While the specific aus line introgressed into Udai21 was not identified in this study, further DNA analysis across aus lines may clarify the exact origins of Udai21. Identifying the specific aus donor would not only clarify why Udai21 maintains high eating quality under heat, but also facilitate the development of new cultivars that leverage the same aus source.

## Conclusion

This study has established a solid foundation for genetic improvement of Udai21 and other rice varieties. By integrating genome assembly, ddRAD-Seq, and resequencing analyses, we successfully constructed a detailed genetic map that facilitated marker-assisted selection and precision breeding. These findings deepen our understanding of Udai21 and provide valuable insights for developing new rice varieties with superior quality traits. Achieving both a high yield and superior eating quality has long been a central objective in rice breeding. However, the emphasis on eating quality has become even more pronounced with rising living standards and increasing consumer demand for high-quality rice [54]. Comparative analysis between Udai21 and Koshihikari revealed differences in gene functions related to resistance and tolerance, physiological traits, and morphological characteristics, which may be attributed to the presence of high- and moderate-impact SNPs (Table 4). For instance, two genes, *UD21ch07g23425* and *UD21ch10g32444*, which encode basic leucine zipper factor 1 and seed-specific protein kinase, respectively, were functionally annotated as genes for eating quality (Table 4). Therefore, missense mutations found in genes between Udai21 and Koshihikari (S1 Table) might confer the eating quality. Further functional analyses (e.g., QTL/association mapping, NILs, and gene validation) are necessary to identify the causative genes responsible for differentiating Udai21 from Koshihikari and elucidate the genetic basis of this cultivar’s unique traits.

## Supporting information

S1 Table Genes with high and moderate impact variants between Udai21 and Koshihikari. (XLSX)

S2 Table Genes that functions were potentially distincted between Udai21 and Koshihikari. (XLSX)
